# Ultrasound-guided percutaneous laser ablation for papillary thyroid microcarcinoma: a retrospective analysis of 37 patients

**DOI:** 10.1186/s40644-019-0204-x

**Published:** 2019-03-20

**Authors:** Lili Ji, Qin Wu, Jun Gu, Xuedong Deng, Wei Zhou, Xing Fan, Feng Zhou

**Affiliations:** 1 Department of Ultrasonography, The Affiliated Infectious Diseases Hospital of Soochow University, 2 Xier Road, Suzhou, 215101 China; 2 Department of Ultrasound, The Affiliated Suzhou Hospital of Nanjing Medical University, No. 1 Lijiang Road, Suzhou, 215163 China; 3 Center for Medical Ultrasound, The Affiliated Suzhou Hospital of Nanjing Medical University, 16 Baita Road, Suzhou, 215001 China; 4 Department of Ultrasound, Rui Jin Hospital, School of Medicine, Shanghai Jiao Tong University, 197 Ruijin 2 Road, Shanghai, 200025 China

**Keywords:** Percutaneous laser ablation, Thyroid micropapillary carcinoma, Ultrasound, Minimally invasive surgery

## Abstract

**Background:**

Over the past ten years, more papillary thyroid microcarcinoma (PTMC) has been diagnosed more frequently due to the development of imaging technology, and the incidence of PTMC has increased significantly. Ultrasound-guided percutaneous laser ablation (PLA) is mainly used for benign thyroid nodules, and few studies have been published on the use of PLA for PTMC. In the present study, a retrospective analysis was performed to explore the efficacy of PLA for PTMC.

**Methods:**

A total of 37 patients with PTMC who underwent PLA were included in this study. Measurement of the lesion volume and serum thyroid hormone levels and clinical evaluation were performed at 1, 3, 6, and 12 months and every 6 months thereafter.

**Results:**

We found that all patients were successfully treated with PLA without serious complications. At the last follow-up visit, 12/37 (32.4%) primary lesions had disappeared, and 24/37 (64.9%) remained as cicatricial hyperplasia. One patient (2.7%) had cervical lymph node metastasis at 24 months post-operatively and underwent open surgery.

**Conclusion:**

Our initial studies suggest that ultrasound-guided PLA is a safe and effective treatment for PTMC.

## Background

Thyroid cancer is the most common endocrine malignancy. Over the past 35 years, the incidence of papillary thyroid cancer (PTC) has tripled, largely due to the increasing incidence of papillary thyroid microcarcinoma (PTMC) [1]. These small cancers that measure 1.0 cm or less account for approximately 50% of the rise in PTC incidence [2]. Controversies still remain regarding thyroid resection and preventive central neck dissection, although PTMC has been shown to be cured by open surgery in the past [3, 4]. However, disease management occasionally becomes a dilemma for patients who are at a high risk for open surgery or are unwilling to undergo surgery [5]. In these circumstances, minimally invasive local therapy has been suggested to be an effective and safe treatment strategy.

In recent years, minimally invasive procedures have been suggested for local control of small-sized neck recurrences of PTMC, which could also reduce possible reoperation morbidity [6]. Ultrasound-guided percutaneous laser ablation (PLA) has been recommended as an effective treatment strategy in the 2010 Thyroid Nodule Guidelines of the European Thyroid Association and American Association of Clinical Endocrinologists; this treatment has been described as an effective thermal ablation technique to treat thyroid nodules and hepatic carcinoma for many years [7–9]. Several trials have demonstrated that a single session of PLA can effectively induce a clinically significant volume reduction in thyroid lesions [10, 11]. The aim of our study was to assess the feasibility, effectiveness, and safety of PLA for PTMC.

## Methods

### Patients

This retrospective study was approved by the Institutional Review Board of Suzhou Hospital Affiliated with Nanjing Medical University (NO.IRB20160601). Informed consent was signed by all patients. A total of 37 patients were treated with PLA for PTMC from May 2016 to April 2017. Of the 37 patients, 12 were men, and 25 were women, with a mean age of 43.9 years (range, 24 to 79 years).

The inclusion criteria for the patients were as follows: (1) a single lesion without coarse calcification and a maximum diameter ≤ 10 mm; (2) all PTMCs diagnosed by fine-needle aspiration biopsy (FNAB); (3) poor surgical candidates and those with high risk of undergoing general anaesthesia; and (4) no history of thyroid surgery or radioiodine therapy.

The exclusion criteria were as follows: (1) multiple lesions or a single lesion with a maximum diameter>1.0 cm; (2) ultrasonography showing enlarged cervical lymph nodes, suspicious metastatic lymph nodes or distant metastasis; (3) large calcifications in the nodules or a diameter of calcifications≥2.0 mm; (4) abnormal function of the vocal cords contralateral to the lesion; or (5) a lesion located in the isthmus or invading into the thyroid capsule, carotid artery or trachea.

### Pre-PLA observation

Patients were evaluated with a real-time ultrasound system equipped with a 13-MHz linear probe and a built-in neodymium yttrium-aluminium-garnet (Nd-YAG) laser device (EchoLaser; Elesta, Florence, Italy). The site, size, volume, and ultrasonographic characteristics of the lesion were evaluated. Laboratory assessment included measurement of serum thyroid stimulating hormone (TSH), free triiodothyronine (FT3), free tetraiodothyronine (FT4), and thyroglobulin (Tg) levels, anti-Tg antibodies, blood cell counts, and prothrombin time. Patients received complete information about the details of treatment.

The volume of the tumour was calculated as follows: *V = Π × a × b × c/6*, where V represents the volume, a represents the length, and b and c represent the other two perpendicular diameters. The mean volume increasing multiplier (MVIM) was calculated as follows: MVIM = (V2-V1)/V1 × 100%, where V2 represents the postoperative volume, and V1 represents the preoperative volume.

### PLA method

**Fig. 1:**
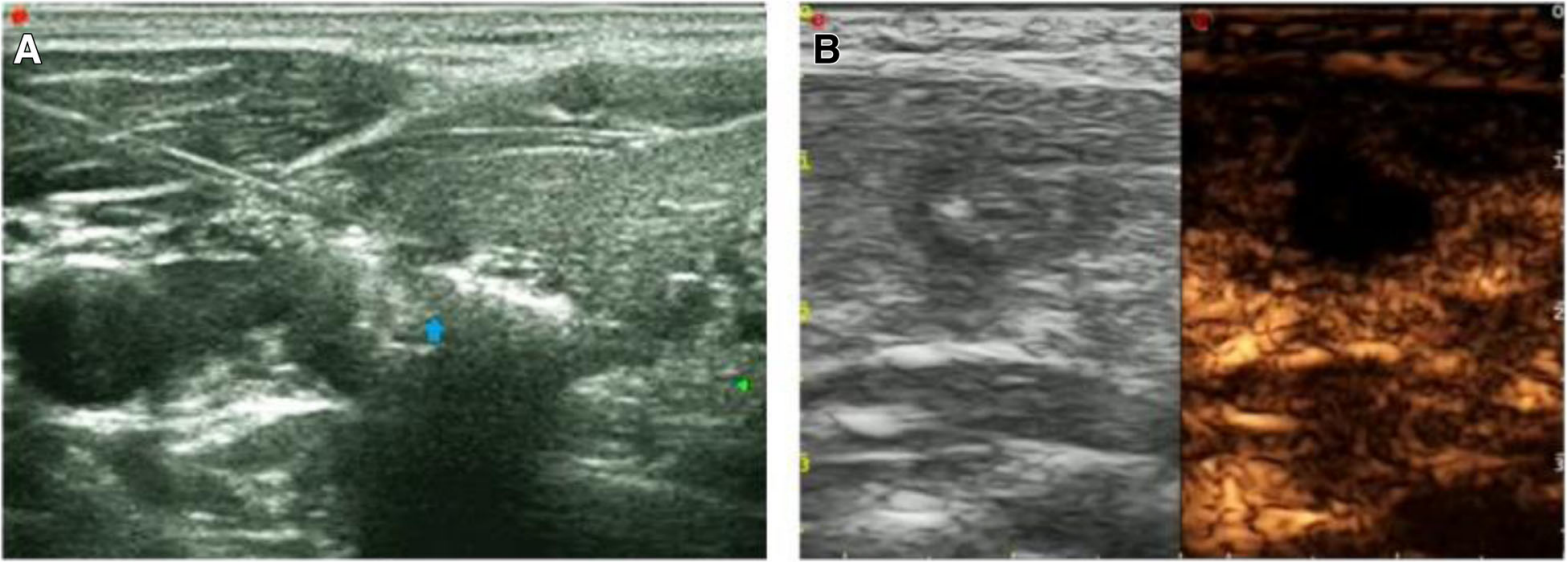
**a** During the course of PLA under ultrasound guidance, the lesion was carbonized with the release of ablation energy, and a hyperechoic area could be observed. **b** CEUS was conducted are the completion of PLA, and a hypoechoic area representing complete ablation could be observed

Patents were placed in the supine position with their necks fully exposed. Local anaesthesia with 2% lidocaine was injected after disinfecting the towel. Depending on the lesion location, hydrodissection solution was used to isolate the important tissue and organs before ablation. The hydrodissection solution was 2% lidocaine plus normal saline (1:8 dilution). After isolation, a 21-G guide needle was used to puncture to the target lesion quickly under ultrasonographic guidance. Then, the stylet was removed, and the fibre was inserted. After that, the guide needle was withdrawn approximately 5 mm, and the fibre head was maintained in the original position directly in contact with the focus. The optic fibre was connected to a continuous-wave Nd:YAG laser source operating at 1.064 mm with an optical beam-splitting device (EchoLaser X4, Esaote, Florence, Italy). Continuous expansion of a strong gas echo area around the fibre head was indicated by the release of energy, and the ablation was stopped once the lesion was completely covered. Then, rapid ablation of the laser ablation needle path was performed (Fig. 1a). To evaluate the ablation situation, the extent of heat damage was observed immediately after the operation. Then, rapid PLA was conducted with an output power of 3 or 4 W. Contrast-enhanced ultrasound (CEUS) was used immediately after PLA (Fig. 1b). When the defect area was completely covered by the thyroid contrast agent filling and the filling exceeded the edge of the primary lesion by 1–2 mm, the lesion was considered completely ablated. Complementary ablation was performed if contrast agent perfusion could be observed.

### Post-PLA observation and follow-up

Complications and tumour volume were recorded. One week after PLA, CEUS was performed to confirm whether the lesions were completely resolved. Additionally, thyroid-related hormone levels were measured, including TSH, FT3 and FT4 levels. If thyroid-related hormone levels were abnormal, the patients were re-examined every month. Regular ultrasound examination of all thyroid glands, in addition to chest radiographs or CT, was performed to monitor for tumour recurrence and cervical lymph node metastasis. The patients were examined after PLA at 1, 3, 6, and 12 months and every 6 months thereafter. The lesion size, blood supply and necrosis were observed using ultrasonography. The volume and the reduction in lesions were evaluated during follow-up.

### Statistical analysis

All statistical analyses were performed using the Statistical Package for the Social Sciences (SPSS) software for Windows 18.0 (SPSS Inc., Chicago, Illinois). All data are expressed as the mean ± standard deviation. The maximum diameter and volume post-PLA were compared with the pre-PLA data with the paired *t-test*. *P* < 0.05 was considered to be statistically significant.

## Results

### General characteristics

All patients underwent PLA for the first time. Of 37 unilateral lesions, 17 were on the left side, and 20 were on the right side. The average volume of hydrodissection solution used during PLA was 10.3 ± 9.7 ml, ranging from 0 to 37 ml. The mean total energy delivery during PLA was 989.4 ± 417.6 J, ranging from 450 J to 1750 J. The active time during PLA was 165.9 ± 92.8 s, ranging from 150 s to 500 s. The average follow-up period was 16.5 ± 6.9 months, ranging from 12 to 24 months. In addition, of the 37 patients, 29 patients were treated with a single needle and single PLA procedure, and 8 patients were underwent a secondary ablation procedure due to incomplete ablation as evidenced by CEUS. They were not found to have metastatic cervical lymph nodes or distant metastasis at the next follow-up visit. Post-operative CEUS demonstrated complete ablation. No contrast agent perfusion was found in the ablation area, and the range was significantly larger than that in the original lesion **(**Table 1**)**.

**Table 1:** Patient characteristics

Characteristics	*N* = 37
N/Sex	F = 25, M = 12
Age (years)	43.9 ± 17.6 (24~79)
PTMC before PLA	
Maximum diameter (mm)	5.1 ± 3.4
Volume (mm^3^)	52.8 ± 30.6
Thyroid hormone levels before PLA	
FT3 (pmol/L)	4.1 ± 1.9
FT4 (ng/dL)	1.6 ± 0.8
T3 (ng/dL)	155 ± 37
T4 (μg/dL)	8.6 ± 3.6
TSH (mU/L)	2.6 ± 1.9
Volume of hydrodissection solution during PLA (mL)	10.3 ± 9.7 (0~37)
Total energy delivery during PLA (J)	989.4 ± 417.6 (450~1750)
Active time during PLA (s)	165.9 ± 92.8 (150~500)
Follow-up (months)	16.5 ± 6.9 (12~24)

*PTMC* papillary thyroid microcarcinoma, *PLA* percutaneous laser ablation, *FT3* free triiodothyronine, *FT4* free tetraiodothyronine, *T3* triiodothyronine, *T4* thyroxine

### Pre-PLA measurement

Thyroid-related hormone levels in all patients were detected, including TSH, FT3 and FT4, which were within the normal range. The maximum lesion diameter was 5.1 ± 3.4 mm, ranging from 2.7 mm to 9.2 mm. The average volume of the lesion pre-PLA was 52.8 ± 30.6 mm^3^, ranging from 19.4 mm^3^ to 102.7 mm^3^ (Table 2).

**Table 2:** Maximum diameter and change in volume before and after PLA treatment

	Pre-PLA	Post-PLA						
		30 min *N* = 37	1 month *N* = 37	3 months *N* = 37	6 months *N* = 35	12 months *N* = 30	18 months *N* = 15	24 months *N* = 8
Maximum diameter (mm)	5.1 ± 3.4	12.9 ± 4.1#	14.7 ± 4.5#	7.9 ± 3.6	4.8 ± 2.7	2.3 ± 1.4*	1.7 ± 0.7*#	1.1 ± 0.6*#
Volume (mm^3^)	52.8 ± 30.6	726.5 ± 187.2#	258.1 ± 97.6#	102.7 ± 39.5#	44.7 ± 24.3	10.2 ± 8.7*	5.7 ± 6.5*#	2.1 ± 1.3*#

*P < 0.05, vs Pre-PLA, # *P* < 0.01, vs Pre-PLA

### Complications and prognosis

During PLA, 91.9% (34/37) of patients felt slight pain and burning sensations of the neck, 33 patients were able to tolerate the whole procedure, and 1 patient received 5.0 mg of dezocine injection. All patients felt self-limiting neck swelling to some extent. Of the 37 patients, 1 patient had a cough and fever on the 7th day post-PLA, which had no relationship to PLA treatment. No cases of dysphonia, neck haematoma formation, surgical site infection or vital organ injury occurred during the perioperative period. However, in 1 45-year-old female patient, her TSH levels were increased, and her FT3 and FT4 levels were slightly decreased during the first and second months post-PLA; these abnormalities recovered spontaneously 3 months later. One 31-year-old female patient was found to have an enlarged cervical lymph node 24 months post-operatively, and cytological analysis suggested metastasis of PTC. This patient then received open surgical treatment. No distant metastasis was found following the next follow-up visit (Table 3).

**Table 3:** Complications and prognosis

Complications or prognosis	N=	Treatment
Pain	34	Most patients were well tolerated and 1 patient received 5 mg of dezocine injection
Self-limiting neck swelling	37	None
Cough and fever	1	Effective symptomatic treatment
Dysphonia	0	–
Neck haematoma	0	–
Local infection	0	–
Vital organ injury	0	–
Serum hormone abnormalities	1	Patient received a re-examination at 1 month and was found to be spontaneously recovered 3 months later
Cervical lymph node metastasis	1	Open surgery
Distant metastasis	0	–

### Post-PLA measurement

**Fig. 2:**
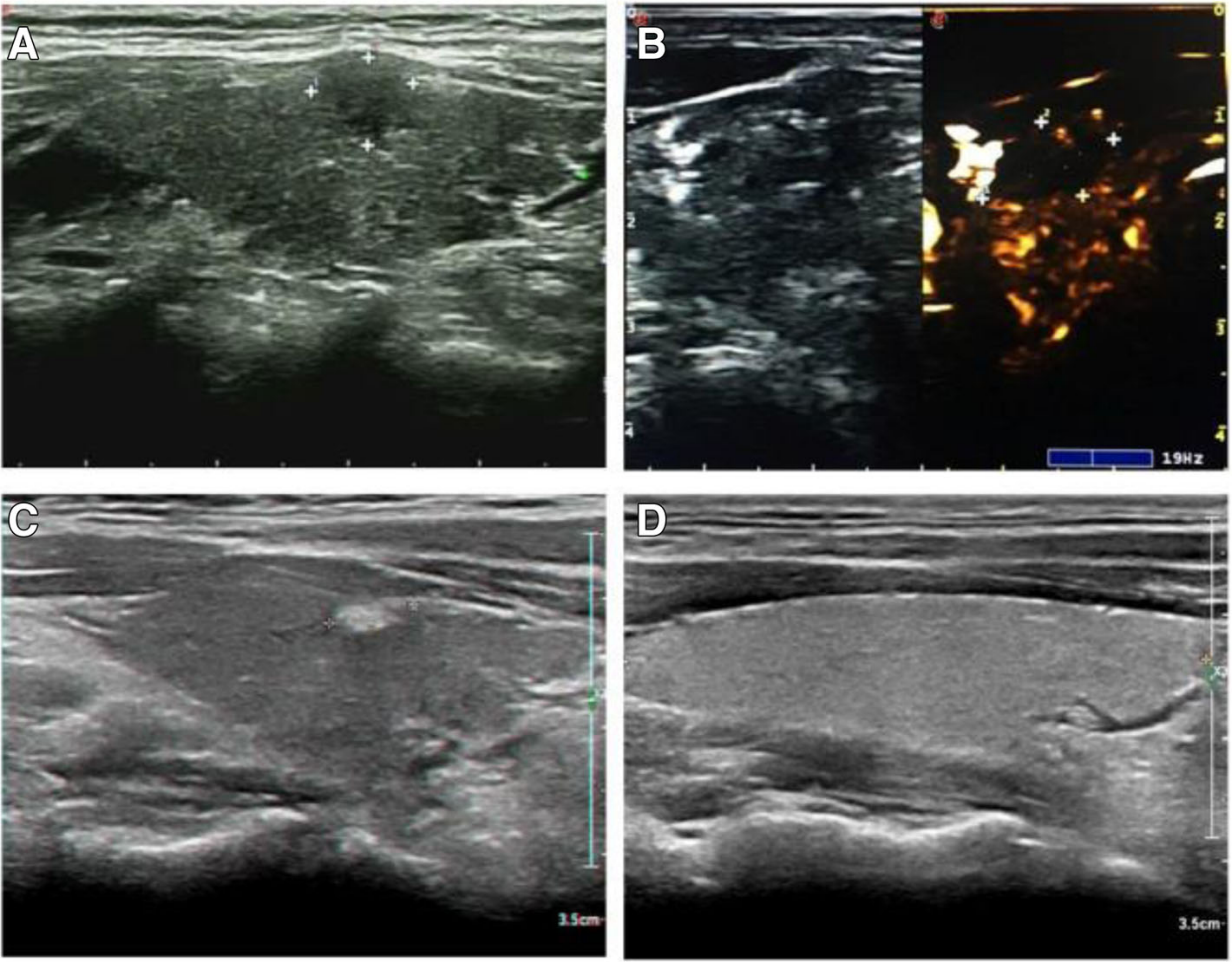
**a** 47-year-old male with a PTMC lesion in whom conventional ultrasound examination showed a 7.8 mm × 6.8 mm × 4.3 mm hypoechoic nodule. **b** After PLA, CEUS revealed a hypoechoic area in the original area, along with some debris in the centre that presented as strong echoes, which demonstrated that the lesion had been completely ablated. **c** Six months after PLA, conventional ultrasound examination showed a 7.1 mm × 3.2 mm × 2.1 mm hyperechoic nodule. **d** Eighteen months after PLA, conventional ultrasound examination showed that the lesion had completely disappeared

**Fig. 3:**
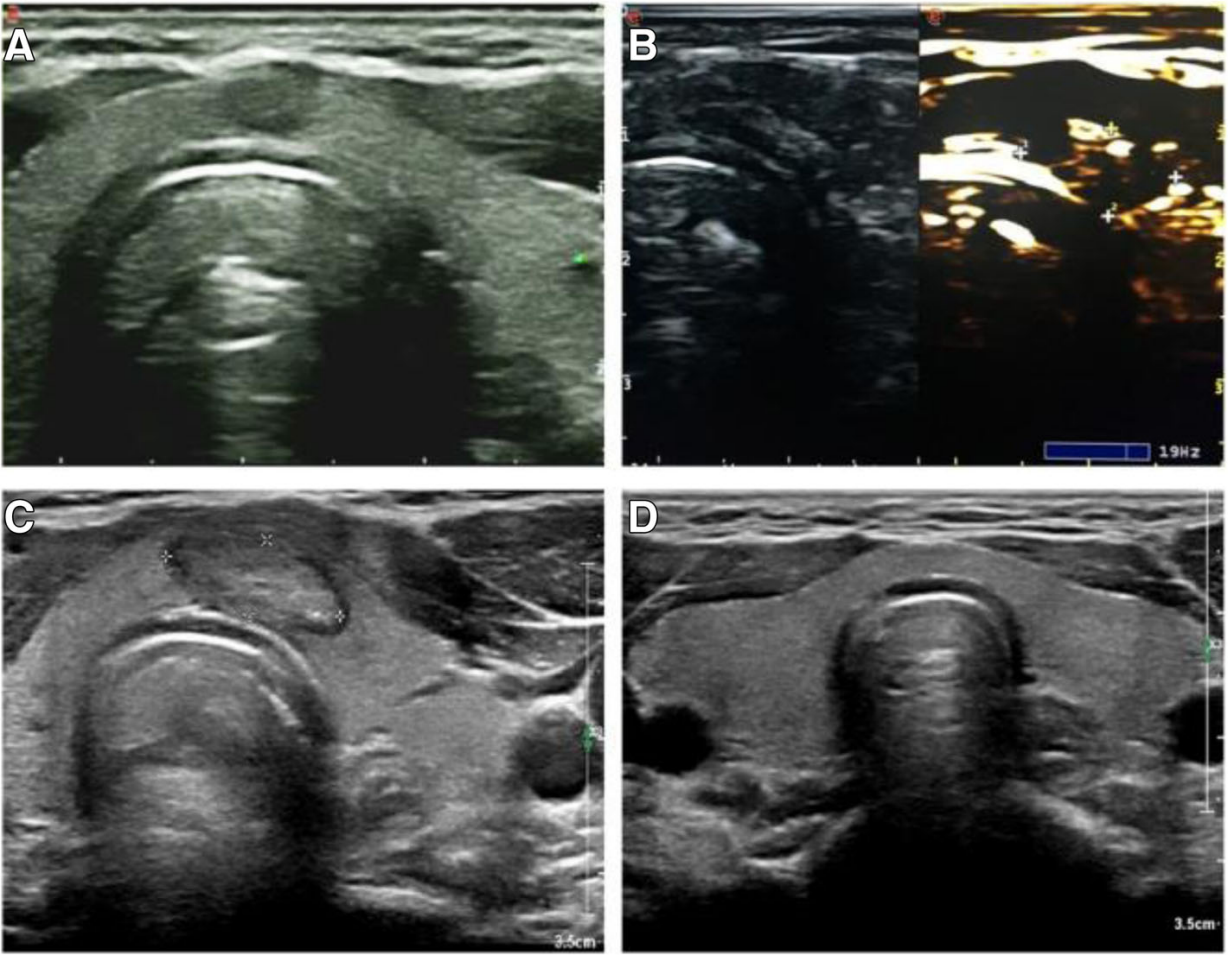
**a** 66-year-old male with a PTMC lesion near the trachea in whom conventional ultrasound examination showed a 9.7 mm × 8.4 mm × 5.5 mm hypoechoic nodule. **b** After PLA, CEUS revealed a hypoechoic area in the original area, and some debris in the centre presented as strong echoes, which demonstrated that the lesion had been completely ablated. **c** One month after PLA treatment, conventional ultrasound examination showed a 15.1 mm × 6.2 mm × 3.3 mm hyperechoic nodule. **d** Eighteen months after PLA treatment, conventional ultrasound examination showed that the lesion had completely disappeared

PTMC presented as a hypoechoic nodule on ultrasound examination preoperatively (Fig. 2a/Fig. 3a). Post-operative ultrasound revealed that with the release of ablation energy, the irregular strong homogeneous gas echo area began to appear around the fibre and gradually expanded along the long axis of the fibre. The lesion became hypoechoic at the end of PLA. A bright echogenic stripe was visible in the centre, which demonstrated that the lesion had been completely ablated, and the boundary between the thyroid parenchyma was clear (Fig. 2b/Fig. 3b). The ablation range was obviously covered by the original swelling and contraction range. During the first month post-PLA, ultrasound showed an enlarged ablation site, which was the combination of the lesion and surrounding tissue (Fig. 3c). During the 6th month post-PLA, ultrasound showed a hyperechoic nodule (Fig. 2c). At the 6-, 12-, 18- and 24-month follow-up visits, ultrasound demonstrated that the ablation area began to shrink gradually (Fig. 2d/Fig. 3d).

**Fig. 4:**
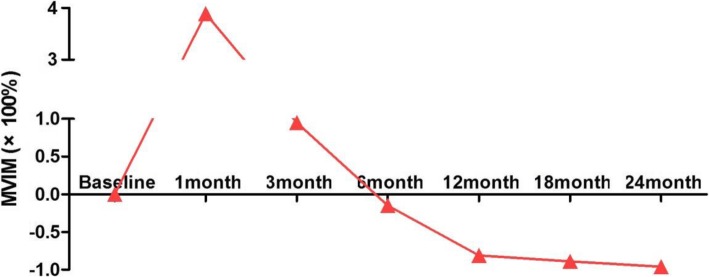
Mean volume increasing multiplier (MVIM) at each follow-up visit after PLA. The MVIM of the tumours was markedly higher than the baseline within 3 months post-PLA due to acute tissue oedema. The MVIM tended to relatively decrease after 6 months, probably due to the gradual absorption of carbonized necrotic tissue

During the follow-up period, the mean maximum diameter and volume at 30 min, 1 month and 3 months were significantly larger than those pre-PLA (Table 2**)**. Moreover, the mean maximum diameter and volume at 12 months, 18 months and 24 months were markedly smaller than those pre-PLA (Table 2**)**. The MVIM revealed that the mean volume within 3 months post-PLA was significantly higher than baseline due to acute tissue oedema, while the MVIM tended to decrease at 6 months and thereafter, most likely due to the gradual absorption of carbonized necrotic tissue (Fig. 4).

The last follow-up evaluation revealed that 12/37 (32.4%) primary lesions had disappeared, and 24/37 (64.9%) remained as cicatricial hyperplasia. Only 1 patient was found to have cervical lymph node metastasis 24 months post-operatively, and this patient received open surgical treatment.

## Discussion

The treatment of PTMC has been controversial for many years [12]. The previously predominant treatment methods for PTMC were surgical resection, endoscopic thyroidectomy, I^131^ treatment or surveillance [5]. In recent years, ultrasound-guided thermal ablation for minimally invasive treatment of PTMC has attracted increasing attention. Thermal ablation is more likely to lead to nodular degeneration and necrosis than percutaneous ethanol injection [13, 14]. PLA is a type of thermal ablation that has been successfully used for the treatment of liver cancer, lung cancer, pancreatic cancer and other tumours [15–17]. Recently, reports have supported the use of PLA for thyroid lesions to achieve non-surgical targeted cytoreduction of tumour cells [18]. PLA of thyroid nodules has several advantages over open surgery: this technique is minimally invasive and effective and does not leave scars on the neck [19]. However, while the use of PLA to reduce the volume of thyroid nodules has been largely reported, very few studies have examined the single-session complete ablation rate for PTMC.

In the present study, the MVIM was 389, 95, − 15%, − 81, − 89% and − 96%, at 1, 3, 6, 12, 18 and 24 months, respectively, after PLA. The ablation enlarged the base of the primary lesion during PLA, and the mean volume of the ablation range was markedly larger than that of the primary lesion at 1 and 3 months after PLA. As the necrotic tissue absorbed, the ablation area gradually decreased, and the MVIM reached 96% at 2 years post-PLA. Only one case of lymph node metastasis occurred in 37 patients, and the patient underwent open surgery. Among the 37 patients, no distant metastasis was found during the follow-up period. At the last follow-up visit, 32.4% of the primary lesions had disappeared, and 64.9% remained as cicatricial hyperplasia, indicating that PLA treatment was effective for PTMC.

Surgical therapy for thyroid disease should be given close attention because the thyroid gland is located adjacent to vital structures, such as the oesophagus, trachea, recurrent laryngeal nerve and carotid artery. PLA seems to be more suited than other treatments for generating a predictable and well-defined area of necrosis for small thyroid lesions close to vital structures of the neck. Valcavi [20] followed up 122 patients with benign thyroid nodules treated with PLA. After 3 years of follow-up, the average size of the lesion in 52.3% of patients was effectively reduced, 1.6% of patients had delayed laryngeal insufficiency after 6–8 weeks, and 3.2% had abnormal thyroid function. In this study, no severe complications, such as massive bleeding or recurrent laryngeal nerve injury, were found in 37 patients after ablation. Only one patient had hypothyroidism, which might have been caused by excessive ablation, and this patient recovered spontaneously 3 months later.

Clinical practice has revealed that the injection of hydrodissection solution and the application of ultrasound monitoring and positioning or CEUS could contribute to the success of PLA and might reduce the incidence of high-power ablation injury to the surrounding tissues [21]. Moreover, the incidence of complications during ablation is also related to the lesion location [22]. Many vital tissues are located in the anterior cervical region, such as the trachea, recurrent laryngeal nerve, carotid artery and parathyroid gland. Special attention should be paid during the ablation of nodules adjacent to these tissues.

Hypothyroidism is a common complication after open surgical treatment. A previous study indicated that the incidence of hypothyroidism after open surgical treatment for PTMC was as high as 75% [23]. The removal of a large amount of normal gland tissues during open surgery might result in a decrease in endogenous hormone secretion, which could directly lead to hypothyroidism [24]. In contrast, PLA, as a minimally invasive method, could significantly reduce the rate of post-operative hypothyroidism [25]. In the present study, 1 of 37 patients was found to have hypothyroidism during the first and second months post-operatively, and this patient recovered spontaneously 3 months later. However, the risks of nodule enlargement and locoregional nodal metastasis are very low in this group. Surveillance alone is another option for PTMC rather than treatment. A comparative study of surgery, non-surgical treatment or surveillance needs to be performed. Several limitations of this study need to be evaluated: (1) The follow-up period was relatively short, and long-term follow-up was needed to evaluate for recurrence and metastasis. (2) Some inevitable residue might have been left even when ultrasound showed that the tumours were completely ablated. Although some diagnostic tools, such as CT, MRI and elastography, have been used to evaluate extrathyroidal tumour extension and lateral lymph node metastasis associated with PTMC, ultrasound is the first modality used to evaluate thyroid nodules in the clinical field [26]. (3) False-positive or false-negative results might occur when using FNAB before PLA. Unal B et al. [27] reported that 46.7% of patients who had previously undergone FNAB were diagnosed with lesions that were malignant or suspicious for malignancy, and 53.3% were concluded to have lesions that were benign or insufficient for diagnosis. In the present study, repeated aspiration biopsy was performed considering sampling errors in cases in which there was any inconsistency between clinical findings and cytological results for PTMC and in patients with suspicious findings on ultrasonography.

## Conclusions

PLA is a rapid, safe, and inexpensive procedure for ablation and is technically feasible for destroying PTMC. The spread of PLA technology for PTMC will widely enrich PTMC treatment strategies. Moreover, PLA can be performed as either an outpatient or a day-hospital procedure. Should PLA be considered the preferred treatment for PTMC?More prospective studies are still needed to confirm the differences in long-term clinical efficacy of PLA and surgical treatment.

## Abbreviations

CEUS: Contrast-enhanced ultrasound
FT3: Free three iodine thyroxine
FT4: Free four thyroid hormone
MVIM: Mean volume increasing multiplier
Nd:YAG: Neodymium yttriumealuminiumegarnet
PLA: Percutaneous laser ablation
PTC: Papillary thyroid cancer
PTMC: Papillary thyroid microcarcinoma
Tg: Thyroglobulin
TSH: Thyroid stimulating hormone
